# COVID-19 Infection: Viral Macro- and Micro-Vascular Coagulopathy and Thromboembolism/Prophylactic and Therapeutic Management

**DOI:** 10.1177/1074248420958973

**Published:** 2020-09-14

**Authors:** Antonis S. Manolis, Theodora A. Manolis, Antonis A. Manolis, Despoina Papatheou, Helen Melita

**Affiliations:** 1 First Department of Cardiology, Athens University School of Medicine, Athens, Greece; 2 Red Cross Hospital, Athens, Greece; 3 Patras University School of Medicine, Patras, Greece; 4 Onassis Cardiac Surgery Center, Athens, Greece

**Keywords:** COVID-19, SARS-CoV-2, venous thromboembolism, deep venous thrombosis, pulmonary embolism, pulmonary arterial thrombosis, arterial thrombosis, coagulopathy, endothelial dysfunction

## Abstract

Coronavirus-2019 (COVID-19) predisposes patients to arterial and venous thrombosis commonly complicating the clinical course of hospitalized patients and attributed to the inflammatory state, endothelial dysfunction, platelet activation and blood stasis. This viral coagulopathy may occur despite thromboprophylaxis and raises mortality; the risk appears highest among critically ill inpatients monitored in the intensive care unit. The prevalence of venous thromboembolism in COVID-19 patients has been reported to reach ∼10-35%, while autopsies raise it to nearly 60%. The most common thrombotic complication is pulmonary embolism, which though may occur in the absence of a recognizable deep venous thrombosis and may be due to *pulmonary arterial thrombosis* rather than embolism, resulting in thrombotic occlusion of small- to mid-sized pulmonary arteries and subsequent infarction of lung parenchyma. This micro-thrombotic pattern seems more specific for COVID-19 and is associated with an intense immuno-inflammatory reaction that results in diffuse occlusive thrombotic micro-angiopathy with alveolar damage and vascular angiogenesis. Furthermore, thrombosis has also been observed in various arterial sites, including coronary, cerebral and peripheral arteries. Biomarkers related to coagulation, platelet activation and inflammation have been suggested as useful diagnostic and prognostic tools for COVID-19-associated coagulopathy; among them, D-dimer remains a key biomarker employed in clinical practice. Various medical societies have issued guidelines or consensus statements regarding thromboprophylaxis and treatment of these thrombotic complications specifically adapted to COVID-19 patients. All these issues are detailed in this review, data from meta-analyses and current guidelines are tabulated, while the relevant mechanisms of this virus-associated coagulopathy are pictorially illustrated.

## Key Points

- Coronavirus-2019 (COVID-19) predisposes patients to arterial and venous thrombosis that raises mortality and is attributed to the inflammatory state, endothelial dysfunction, platelet activation and blood stasis
- The most common pattern is *pulmonary arterial thrombosis* resulting in thrombotic occlusion of small- to mid-sized pulmonary arteries and subsequent infarction of lung parenchyma
- Biomarkers related to coagulation, platelet activation and inflammation have been suggested as useful diagnostic and prognostic tools for COVID-19-associated coagulopathy; among them, D-dimer remains a key biomarker employed in clinical practice
- Current guidelines or consensus statements can guide thromboprophylaxis and treatment of these thrombotic complications specifically adapted to COVID-19 patients

## Introduction

Coronavirus-2019 (COVID-19) has been notorious for the pulmonary and cardiovascular complications that it confers.^1-4^ However, more recently, this novel virus, responsible for an unprecedented pandemic disease and the world-wide turmoil that it has created, has also been demonstrated to predispose patients to arterial and venous thromboses attributable to the inflammatory state, platelet activation, endothelial dysfunction, and blood stasis.^5^

A *viral coagulopathy* correlating with poor prognosis has been amply described in recent reports in patients with COVID-19 infection who may exhibit pulmonary embolism (PE); venous, arterial, and microvascular thrombosis; lung endothelial injury; and associated thrombotic complications leading to and/or worsening acute respiratory distress syndrome (ARDS).^6^ D-dimers and fibrin/fibrinogen degradation products are particularly elevated in these patients.^7^ However, this coagulopathy is not characterized by consumption of coagulation factors, as seen in disseminated intravascular coagulation (DIC).^8^

**Figure 1.**
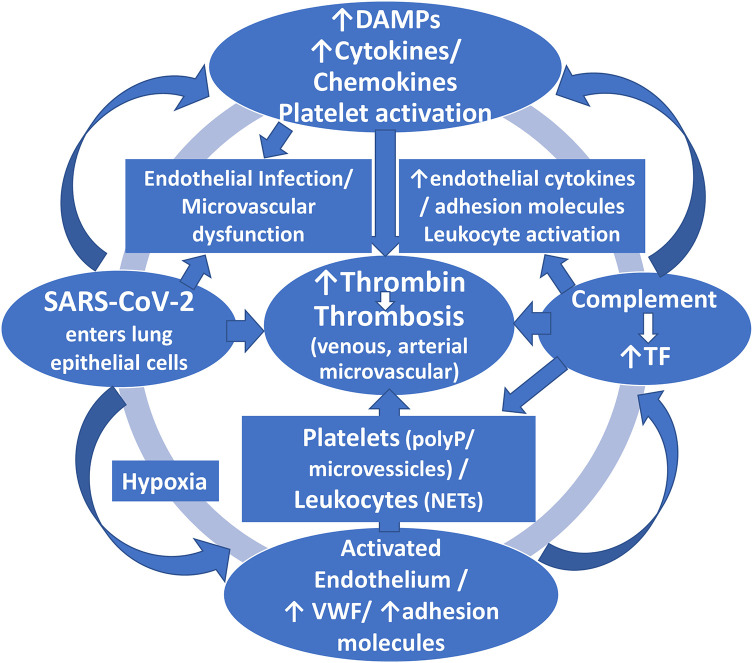
The schema illustrates the proposed mechanisms of SARS-Cov-2-induced coagulopathy. The virus not only enters the host lung epithelial cells but can invade endothelial cells, as well. Infection of host cells leads to the release of damage- or danger-associated molecular patterns (DAMPs) (host biomolecules that can initiate and perpetuate a noninfectious inflammatory response by activating the innate immune system), and also the release of proinflammatory cytokines and chemokines. Furthermore, leukocytes and platelets are recruited and activated that finally lead to initiation of intravascular thrombin generation which further activates endothelial cells, platelets and leukocytes in a continuous feedback loop that perpetuates thrombin generation and thrombosis. In this cascade, complement activation also plays a prothrombotic role by recruiting leukocytes and amplifying platelet activation and enhancing endothelial dysfunction and proinflammatory actions. The hypoxic milieu can further enhance these processes. This thrombotic cascade finally leads to clinical manifestations of this viral coagulopathy that include deep vein thrombosis, pulmonary embolism, arterial thrombosis, microvascular thrombosis and ischemic stroke. NETs = neutrophil extracelluar traps; PolyP = polyphosphate; TF = tissue factor; vWF = Von Willebrand factor.

The molecular mechanisms responsible for the hypercoagulable state observed in patients with COVID-19 are still incompletely elucidated; however, there appears to be a close link between inflammatory and hemostatic systems (Figure 1).^9,10^ Current data suggest that COVID-19 can infect endothelial cells with an ensuing associated immune response and attendant activation of inflammatory pathways resulting in dysregulation of the endothelium, leukocyte activation, neutrophil extracellular traps (NET) generation (a matrix of DNA decorated with neutrophil granule proteins, such as myeloperoxidase, cathepsin G, and neutrophil elastase), complement deposition, and platelet activation and consumption.^10^ It has been suggested that the virus instigates the process of *pyroptosis*, an inflammatory form of programmed cell death observed upon infection with intracellular pathogens, which could contribute to the endothelial cell death after COVID-19 infection and could increase proinflammatory cytokine releases, such as interleukin (IL)-1 beta and IL-18.^11^ Both these pathologic processes of endothelial dysfunction and pyroptosis might lead to systemic thrombotic events.^11^ Monitoring of coagulation indices in severely ill COVID-19 patients seems imperative to identify those patients at increased thromboembolic risk and to adjust thromboprophylaxis accordingly.

Furthermore, there are some data for *complement* participating in the pathogenesis of thrombosis and end-organ damage in these patients.^12^ It has been proposed that SARS-CoV-2 may also trigger complement activation through its recognition by the host as a foreign pathogen, by acting as a cofactor to enhance lectin pathway activation, and by invading and injuring ACE2 receptor-expressing host cells, with all these actions promoting a thromboinflammatory response, which in turn, re-circles to further amplify complement activation and clotting.^12^ Hence the suggestion that early intervention for COVID-19 with anticomplement agents may be important to limit cell/tissue damage, which remains to be tested (see discussion below). As mentioned, in addition to complement, *neutrophils* yielding high tissue factor (TF) expression and releasing NETs carrying active TF, also contribute to the maladaptive immune response that leads to hyper-inflammatory reaction and thrombotic microangiopathy; hence, the recommendation for strategies against SARS-CoV-2 that exploit complement and/or NET inhibition.^10,13^ Finally, *hypoxia* has been suggested as a potent trigger of thrombosis in COVID-19 that may also offer an explanation of the apparent resistance of COVID-19 patients to standard dose of heparin for effective thromboprophylaxis.^14^

## Venous Thromboembolism (VTE)/Deep Vein Thrombosis (DVT)/Pulmonary Embolism (PE)/Pulmonary Arterial Thrombosis

Venous thromboembolism (VTE) very commonly complicates the clinical course of inpatients with COVID-19, despite thromboprophylaxis; the risk appears highest among critically ill inpatients monitored in the intensive care unit (ICU).^15^ The incidence of VTE in COVID-19 patients has been reported to occur in ∼10-35%, with autopsies indicating that it may reach nearly 60%^12,16^; with pulmonary embolism being the most common thrombotic complication.^17^ A recent large meta-analysis of 44 studies reporting on acute complications and mortality in 14,866 hospitalized COVID-19 patients indicated that VTE occurred in 15%; however, the authors admit to very low-quality evidence due to high heterogeneity and risk of bias (Table 1).^18^

**Table 1.** Meta-Analyses of Observational Studies Reporting on Thrombotic Complications in Patients With COVID-19 Infection.

**Author/Year**	**No of Studies/** **Patients**	**VTE**	**PE**	**DVT**	**Comments**
Potere et al / 2020^18^	44 / 14,866	15%			N.B.: The number of studies reporting on VTE is limited to only 3 studies (318 patients)
Wang et al / 2020^19^	28 / 4138			16%	Pooled DVT prevalence 23% in patients treated in ICU vs 5% in patients treated in non-ICU (*P* < 0.01); 30% in patients from China vs 13% in those from western countries (*P* < 0.01)
Chi et al / 2020^20^	11 / 1981	23.9%	11.9%	11.9%	VTE incidence in ICU setting: 30.4% vs 13% in ward setting PE: 15.7% in ICU vs 2.4% in ward DVT: 10.6% in ICU vs 13.6% in ward
Hasan et al / 2020^21^	12 / 899	31%			All patients were in ICU receiving prophylactic or therapeutic anticoagulation
Fontana et al / 2020^15^	11 / 1369	4.4-8.2% (all patients /3 studies) 0-35.3% (ICU patients /6 studies)			Much greater risks in ICU patients, up to 53.8% N.B.: these numbers occurred despite thromboprophylaxis

DVT = deep vein thrombosis; ICU = intensive care unit; PE = pulmonary embolism; VTE = venous thromboembolism.

A recent systematic search of 28 articles reporting 397 deep vein thrombosis (DVT) cases in a total of 4138 COVID-19 patients indicated that the pooled estimate of the prevalence for DVT was 16% by using a random-effects model.^19^ According to patients’ geographic location, a much higher pooled prevalence of DVT was found in COVID-19 patients from China (30%) compared with those from Western countries (13%, *P*  <  0.01). The pooled prevalence of DVT in COVID-19 patients admitted to the ICU was much higher (23%) compared to COVID-19 patients not requiring ICU monitoring (5%, *P* <  0.01).

A recent study of 184 ICU patients with COVID-19 pneumonia of whom 23 died (13%), 22 were discharged alive (12%) and 139 (76%) were still in the ICU, all receiving thromboprophylaxis, indicated that the cumulative incidence of the composite outcome (PE, DVT, ischemic stroke, myocardial infarction or systemic arterial embolism) was 31%.^17^ Computed tomography (CT) pulmonary angiography (CTPA) and/or ultrasonography confirmed VTE in 27% and arterial thrombotic events in 3.7%, while PE was the most frequent thrombotic complication (n = 25, 81%). Age (adjusted hazard ratio - aHR 1.05/per year) and coagulopathy, defined as spontaneous prolongation of the prothrombin time > 3 s or activated partial thromboplastin time (aPTT) > 5 s (aHR 4.1), were independent predictors of thrombotic complications.

Another study of 101 COVID-19 in-hospital patients undergoing duplex ultrasound (DUS) for clinically suspected DVT indicated that 42 were positive for DVT, 7 for superficial thrombophlebitis and 24 for PE, 8 of which associated with a DVT.^22^ Time of onset varied greatly, but diagnosis was more frequent in the first 2 weeks since hospital admission (73.8%). Most PEs involved the most distal pulmonary vessels, and two thirds occurred in absence of a recognizable DVT, suggesting a primary thrombosis rather than embolism.^22^

A single-center review of 214 CTPA studies in 1477 patients with suspected or confirmed COVID-19 indicated that the diagnostic yield for PE was 37%.^23^ The overall proportion of PE in patients with COVID-19 was 5.4%. The proportions with Wells score of ≥4 (“PE likely”) was 33/134 (25%) without PE vs 20/80 (25%) with PE (*P* = 0.951). The median National Early Warning-2 (NEWS2) score (illness severity) was 5 in the PE group vs 4 in those without PE (*P* = 0.133). D-dimer was higher in PE (median 8000 ng/mL) than non-PE (2060 ng/mL, *P* < 0.001). In the “low probability” group, D-dimer was higher (*P* < 0.001) in those with PE but had a limited role in ruling out PE. The authors concluded that in a non-critical care setting, PE in hospitalized patients with COVID-19 is common with almost half of PE events diagnosed upon hospital admission.

Among 25 critically ill COVID-19 patients admitted to the ICU, DVT screening at days 5-10 of admission yielded a 32% prevalence of VTE; proximal DVT was detected in 6 (24%), and PE in 5 (20%).^24^ The majority of events (75%) occurred before screening, suggesting a need for earlier screening.

A meta-analysis of 11 cohort studies indicated that among hospitalized COVID-19 patients, 23.9% developed VTE despite anticoagulation.^20^ PE was detected in 11.6% and DVT in 11.9% of patients. Patients in the ICU had a higher risk for VTE (30.4%) than those in the ward (13%). Patients who developed VTE had higher D-dimer levels compared with those who did not develop VTE (mean difference, 2.05 µg/mL; *P* = 0.02). Another meta-analysis of 12 studies where all patients were receiving low-molecular weight (LMWH) or unfractionated heparin (UFH) for thromboprophylaxis, showed that the pooled prevalence of VTE among ICU patients was 31% (95% CI 20-43%).^21^

As alluded to in the above-mentioned studies, VTE can occur in non-critically ill patients with COVID-19 infection. According to a retrospective cohort study of 289 patients (mean age 62.2 ± 17.0 years, 59% male) admitted to general wards with confirmed COVID-19, VTE imaging tests were performed in 100 patients (34.6%) and VTE was detected in 49 patients (17%); PE was diagnosed in 42 patients (14.5%), cerebral venous thrombosis in 3 patients (1%) and DVT in 12 patients (4.2%).^25^ The composite of death or transfer to ICU occurred in 90 patients (31%) and was almost 2-fold higher in VTE patients (47.9% vs 27.9%, *P* = 0.01). Lack of thromboprophylaxis was a major determinant of VTE in non-ICU COVID-19 patients.

Importantly, several data indicate that the majority of diagnosed “PE” cases occur in the absence of a recognizable DVT and may be due to primary in-situ thrombosis (*pulmonary arterial thrombosis*) rather than embolism, resulting in thrombotic occlusion of small- to mid-sized pulmonary arteries and subsequent infarction of lung parenchyma (see discussion below).^22,26,27^ In this context, thrombosis of small and mid-sized pulmonary arteries was found in various degrees in 11 deceased patients with COVID-19,^26^ while in another series, histologic analysis of pulmonary vessels in 7 lungs obtained during autopsy from patients who died from COVID-19, showed widespread thrombosis with microangiopathy; alveolar capillary microthrombi were 9-fold more prevalent in patients with COVID-19 compared with patients with influenza (*P* < 0.001).^28^

## Other Systemic Thrombosis/Thromboembolism

Case reports of systemic thrombosis have recently been published, such as cerebral venous thrombosis in a young woman with COVID-19 infection,^29^ acute ophthalmic artery occlusion in a young patient with COVID-19 infection despite receiving apixaban for DVT,^30^ femoral artery thrombosis in a young patient with non-severe COVID-19 infection,^31^ and acute superior mesenteric artery thrombosis causing acute intestinal ischemia.^32^ Even a case of heparin-induced thrombocytopenia (HIT) with evidence of thrombosis (lung, upper extremity, skin) was recently reported.^33^ Peripheral arterial embolism has occasionally been reported as the initial presentation of patients with COVID-19 infection.^34^

A recent study comparing 16 patients with COVID-19 (age 70 ± 14 years, 7 women) undergoing lower extremity CT angiogram (CTA) with 32 propensity-score matched control patients (age 71 ± 15 years, 16 women), showed that all COVID-19 patients had at least 1 arterial thrombus while only 69% of controls had arterial thrombi (*P* = 0.02); proximal thrombi were present in 94% of COVID-19 patients compared with 47% of controls (*P* < 0.001).^35^ Death or limb amputation was more common in COVID-19 patients (odds ratio-OR 25, *P* < 0.001).

Furthermore, recent data from 115 patients presenting with ST-elevation myocardial infarction (STEMI) and concurrent COVID-19 infection indicated that there was a strong trend toward higher thrombus burden and poorer outcomes.^36^ Interestingly, other data from a recent case of STEMI have indicated the presence of microvascular thrombi accounting for the dismal outcome of this STEMI patient in the absence of epicardial vessel occlusion.^37^ Another case of STEMI in a COVID-19 patient complicated by endocavitary thrombus was recently described despite receiving anticoagulation therapy; D-dimer levels were inordinately elevated and the patient succumbed to his disease.^38^ A case of multiple coronary thromboses causing STEMI in a patient with COVID pneumonia was reported who was managed with thromboaspiration, coronary stenting and combined antiplatelet and anticoagulant therapy.^39^ Finally, a high rate of stent thrombosis (4/19 or 21%) was recently reported in COVID-19 patients presenting with STEMI managed with primary percutaneous coronary intervention (PCI), indicating a possible need to modify STEMI management for COVID-19 patients.^40^

Importantly, pathological data indicate that thrombosis appears to be a prominent feature in multiple organs, in some cases despite full anticoagulation and regardless of timing of the disease course, as suggested by the finding of megakaryocytes and platelet-rich thrombi in the lungs, heart and kidneys.^41^

## Hypercoagulable State

The association of COVID-19 infection severity with changes in hemostatic parameters was explored in a meta-analysis of 60 studies comparing 5487 patients with severe and 9670 patients with mild COVID-19 infection.^42^ The meta-analysis found a higher prothrombin time (PT) (standardized mean difference-SMD: 0.41), D-dimer (SMD: 0.67), and fibrinogen values (SMD: 1.84), with lower platelet count (SMD: -0.74) among severe infection patients. Analysis of 25 studies on 1511 COVID-19 non-survivors and 6287 survivors showed higher PT (SMD: 0.67) and D-Dimer values (SMD: 3.88), with lower platelet count (SMD: -0.60) among non-survivors. Regression models showed that C-reactive protein (CRP) values were correlated with the difference in PT and fibrinogen levels.

A recent systematic review of 16 studies pointed to existing correlations between COVID-19 infection, severe elevation of D-dimer levels, and increase in the rate of complications.^43^ Such findings suggest a significant role of early and continuous D-dimer monitoring and labeled anticoagulation as management tools for COVID-19 disease to prevent complications and reduce interventions, as COVID-19 patients treated with anticoagulants demonstrated lower mortality compared with those not treated (*P* = 0.017).

## Impaired Fibrinolysis

COVID-19 patients present not only with hypercoagulability but also with impaired fibrinolysis. Fibrinolytic activity and thrombin generation were assessed in 78 COVID-19 patients, of whom 48 were admitted to the ICU and 30 to the medical ward.^44^ All patients received thromboprophylaxis with heparin. A high thrombin generation capacity was observed, which was not mitigated with heparin therapy; furthermore, a hypofibrinolytic state was observed, mainly associated with increased plasminogen activator inhibitor-1 (PAI-1) levels. Similarly, another study of 21 COVID patients in the ICU who had a rotational thromboelastometry test, indicated that 11 (52.4%) met the criteria for impaired fibrinolysis.^45^ Nine (42.9%) patients had been diagnosed with VTE. Eight (89%) of these 9 VTE patients met criteria for impaired fibrinolysis. The authors concluded that these data support the use of viscoelastic testing to evaluate for the presence of impaired fibrinolysis to identify patient subsets who might benefit from the administration of fibrinolytics.

## Thromboembolism Versus Microthrombosis

As already mentioned, there seem to be 2 phenotypic patterns of thrombotic manifestations of COVID-19 infection, the classical *thromboembolic* disease, also observed in other types of sepsis, and the diffuse *micro-thrombotic* type, prevailing in the lungs but occasionally extending to other organs, as well.^26,46^ Both types can cause severe disease and are potentially lethal. The micro-thrombotic pattern seems more specific for COVID-19; it is characterized by hypercoagulability associated with an intense immuno-inflammatory reaction that results in diffuse occlusive thrombotic micro-angiopathy with alveolar damage and vascular angiogenesis. Coexisting impaired fibrinolysis exacerbates the thrombotic process and leads to persistence of micro-thrombi.^46^

The role of vascular angiogenesis as a distinctive feature of COVID-19 infection was recently demonstrated in a small pathology series, where vascular angiogenesis distinguished the pulmonary pathobiology of COVID-19 from that of equally severe influenza virus infection; the lungs from patients with COVID-19 had widespread vascular thrombosis with microangiopathy and occlusion of alveolar capillaries.^28^ Similar findings were reported by another pathology series, where thrombosis of small and mid-sized pulmonary arteries was found in various degrees in 11 deceased patients with COVID-19 and was associated with infarction in 8 patients and bronchopneumonia in 6 patients.^26^

## Pulmonary Angiopathy/Computed Tomography Findings

A recent study comprising 39 consecutive patients with COVID-19 pneumonia (32 males, age 53 ± 10 years, 64% black and ethnic minority) undergoing CTPA and/or dual-energy CT showed that there was a significant vascular perfusion abnormality and increased physiologic dead-space with evidence of hypercoagulability and fibrinolytic suppression.^47^ Perfusion defects on CT (assessable in 18/20 [90%]) were present in all patients. The authors concluded that these data show not only the presence of a hypercoagulable phenotype in severe COVID-19 pneumonia but also markedly impaired pulmonary perfusion likely caused by pulmonary angiopathy and thrombosis. Similarly, among 34 COVID-19 patients who underwent CTPA, 26 had PE (76%; 20 males, median age 61 years, 77% with comorbidities).^48^ Mortality was at 31% in this group. Importantly, 8 PE patients had been under thromboprophylaxis with low-molecular-weight heparin; 4 PE patients had DVT at ultrasound examination.

Another study of 51 patients (mean age 45 years, 74.5% men) with proven COVID-19 pneumonia receiving extracorporeal membrane oxygenation (ECMO) investigated the presence and extension of pulmonary thromboembolic disease via chest CT.^49^ All patients had severe COVID-19 pneumonitis, and 18/51 (35.3%) had macroscopic thrombosis (15 with associated ischemia); however, 13/51 (25.5%) patients had ischemia without associated thrombus, highly suggestive of lung ischemia due to isolated microvascular immune thrombosis.

## Markers of Thrombosis

Clinical criteria are of utmost importance in rendering a diagnosis of VTE, which are greatly enhanced with use of D-dimer levels.^50^ As mentioned, multi-detector CTPA to diagnose PE and compression ultrasound to diagnose DVT are the preferred imaging modalities. The development of PE-excluding algorithms are most important, e.g. applying the YEARS diagnostic algorithm in patients with suspected PE which includes 3 clinical items (clinical signs of DVT, hemoptysis, and whether PE is the most likely diagnosis) and D-dimer levels, PE can be safely excluded.^51^ In this context, several biomarkers related to coagulation, platelet activation and inflammation have been suggested as useful prognosticators of the occurrence of thrombotic complications in patients with COVID-19 infection (Table 2). Among them, D-dimer levels seem to be the strongest predictor.^52^

**Table 2.** Markers of Thrombosis in Patients With COVID-19 Infection.

Coagulation Markers
D-dimer (the most useful marker)
Fibrinogen
Fibrin/fibrinogen degradation products
von Willebrand Factor
PT/APTT
Platelet count
Platelet Activation
Thromboxane B2
P-selectin
Soluble CD40 ligand
Mean platelet volume
Inflammation Markers
Very high CRP
High ESR
Ferritin
Procalcitonin

APTT = activated partial thromboplastin time; CRP = C-reactive protein; ESR = erythrocyte sedimentation rate; PT = prothrombin time.

A recent retrospective study of 158 COVID-19 patients undergoing venous duplex ultrasound testing which rendered a DVT diagnosis in 52 patients showed that all patients had increased D-dimer levels using conventional criteria, and 154/158 (97.5%) had increased levels with age-adjusted criteria (mean D-dimer 16,163 ± 5,395 ng/mL).^52^ Those with DVT had higher D-dimer levels than those without DVT (median 13,602 vs. 2,880, *P* < 0.001); an optimal D-dimer cutoff of 6,494 ng/mL could differentiate those with and without DVT (sensitivity 80.8%, specificity 68.9%, negative predictive value 88.0%). Wells DVT criteria were not found to be a significant predictor of DVT. The authors concluded that D-dimer levels are uniformly elevated in COVID-19 patients; a D-dimer of <6,494 ng/mL may exclude DVT, thus potentially limiting the need for venous duplex ultrasonography.

Another study indicated that high D-dimer levels at initial presentation were predictive of coagulation-associated complications during hospitalization (D-dimer >2500 ng/mL, adjusted odds ratio - OR for thrombosis 6.79, for bleeding 3.56), critical illness, and death.^53^ Additional markers at initial presentation predictive of in-hospital thrombosis included platelet count >450 × 109/L (adjusted OR, 3.56), C-reactive protein (CRP) >100 mg/L (adjusted OR, 2.71), and erythrocyte sedimentation rate (ESR) >40 mm/h (adjusted OR, 2.64). ESR, CRP, fibrinogen, ferritin, and procalcitonin were higher in patients with thrombotic complications than in those without.

A study of 184 COVID patients in the ICU setting indicated that spontaneous prolongation of the prothrombin time (PT) > 3 s or activated partial thromboplastin time (aPTT) > 5 s (adjusted hazard ratio-aHR 4.1, 95%CI 1.9-9.1), were independent predictors of thrombotic complications.^17^

Another study of 100 hospitalized patients with COVID-19 (median age 65 years) proposed that certain biomarkers of platelet activation are associated with thrombosis or death in patients hospitalized with COVID-19.^54^ Specifically, plasma levels of thromboxane B2 (TxB2) (*P* = 0.006), P-selectin (*P* = 0.005), soluble CD40 ligand (sCD40 L) (*P* = 0.016) and mean platelet volume (MPV) (*P* = 0.012) were independently associated with the composite of thrombosis or death. Of the 14 patients who experienced a thrombotic event, only TxB2 was associated with thrombosis after multivariable adjustment (*P* = 0.013). Of the 24 deceased patients, TxB2 (*P* = 0.006), P-selectin (*P* = 0.005), sCD40 L (*P* = 0.016) and MPV (p = 0.012) were associated with all-cause mortality after multivariable adjustment.

## Therapeutic Interventions

As thrombotic complications are key determinants of the high mortality rate in patients with COVID-19 infection, strategies of thromboprophylaxis are of paramount importance to combat these potentially lethal complications. Several antithrombotic agents have been proposed as potential therapies to prevent COVID-19-associated thrombosis, including, low-molecular weight heparin (LMWH) or unfractionated heparin (UFH), direct oral anticoagulants (DOACs), antiplatelet agents, FXII inhibitors, thrombolytic drugs, and nafamostat, many of which also possess pleiotropic anti-inflammatory or anti-viral effects.^10^

However, VTE has been reported despite seemingly adequate thromboprophylaxis. Hence an intensified antithrombotic therapy has been suggested. However, bleeding complications of such intensified antithrombotic therapy may lurk. According to a multicenter retrospective study of 400 hospital-admitted COVID-19 patients (144 critically ill) primarily receiving standard-dose prophylactic anticoagulation, VTE rate was 4.8%, and the overall thrombotic complication rate was 9.5%.^53^ The overall and major bleeding rates were 4.8% and 2.3%, respectively. In the critically ill, VTE and major bleeding rates were 7.6% and 5.6%, respectively. Disseminated intravascular coagulation (DIC), thrombocytopenia, and reduced fibrinogen were rare and were associated with significant bleeding manifestations. The authors concluded that given the observed bleeding rates, randomized trials are needed to determine any potential benefit of intensified anticoagulant prophylaxis in COVID-19 patients.

For cases of documented or highly suspected VTE, therapeutic anticoagulation is the mainstay of VTE management.^5^ When selecting the appropriate antithrombotic agent, one needs to take into account coexisting comorbidities such as renal or hepatic dysfunction, thrombocytopenia, and gastrointestinal function. In general, parenteral anticoagulation (e.g., UFH) is the treatment of choice. However, due to the need for frequent blood drawing to assess the aPTT, LMWHs may be preferred. DOACs may have several advantages as there is no need for follow-up testing and are more convenient to receive both in the hospital and out-hospital setting, although there may be some concern about the prompt availability of effective reversal agents. Catheter-directed therapies are available but should be limited for the most urgent situations. Selective use of inferior vena cava filters is also recommended, especially in the setting of contraindications to anticoagulation.^5^ In cases of submassive PE, rescue systemic fibrinolysis should be considered, with catheter-directed options as an alternative option.^55^ For patients with hemodynamic instability, systemic fibrinolysis or catheter-based therapies are available. Interventional approaches such as aspiration thrombectomy may remove or reduce thrombus material and improve flow in the pulmonary arteries and produce clinical improvement in patients with massive or submassive PE.^56^ In certain critical situations, bedside insertion of extracorporeal membrane oxygenation (ECMO) may be employed.^57^

A retrospective study reported the results of thrombolysis with use of alteplase in 12 COVID-19 patients (median age 61.5 years) with profound hypoxia who failed proning and were on mechanical ventilation (n = 11) or continuous positive air way pressure (n = 1), with or without evidence of pulmonary thrombosis on pulmonary angiography (CTPA).^58^ Five (41.7%) patients had multiorgan failure and required renal replacement therapy. PaO2/FiO2 ratios (PF ratio) pre and 24 h after thrombolysis showed significant improvement in all patients (*P* = 0.002). Seven patients survived to hospital discharge, while the other 5 died (41.7%) from 2-11 days following thrombolysis due to multiorgan failure. Twenty-four hours after thrombolysis, median fibrinogen fell from 7.0 g/L to 3.40 g/L (*P* = 0.03) and median d-dimer increased from 3502 ng/ml to 19450 (*P* = 0.002). There were no major or clinically significant minor bleeding complication of thrombolysis, except for 1 patient who had intracranial bleeding 17 days after thrombolysis while on unfractionated heparin.

Several ongoing RCTs aim to determine the optimal anticoagulation regimen in both ICU and non-ICU patients with COVID-19 infection (CORRIMMUNO-COAG, NCT04344756: active anticoagulation vs standard of care including thromboprophylaxis; COVID-HEP, NCT04345848: therapeutic anticoagulation vs thromboprophylaxis).

Due to the involvement of complement and neutrophils, in addition to platelets and other coagulation and endothelial factors, in the pathogenesis of virus-induced immuno-inflammatory coagulopathy and thrombotic microangiopathy, there is a suggestion that early intervention with anticomplement agents and NET inhibition may be important to limit cell/tissue damage and its attendant thrombosis; however, this remains to be tested.^10,12,13^

A strong argument has also been put forth for the inhibition of interleukin (IL)-1 as a therapeutic strategy to circumvent the deleterious effects of this cytokine which is involved in inducing inflammation, endothelial dysfunction and microthrombi.^59^ All these effects together with the activation of macrophages by COVID-19 lead to the release of pro-inflammatory cytokines, metalloproteinases and other proteolytic enzymes that can cause thrombi formation and severe respiratory dysfunction. IL-1 induces itself and tissue necrosis factor (TNF), which may also participate in hemodynamic compromise and produce shock syndrome in COVID-19, while these pro-inflammatory cytokines can cause pulmonary edema, thrombosis and bleeding, and can produce leukopenia and thrombocytopenia. IL-1 contributes to the formation of thrombi also by stimulating the formation of thromboxane A2 which is released into the inflamed environment; furthermore, IL-1 induces the release of thromboxane B2 (TxB2) in activated neutrophils and macrophages, and stimulates endothelial cell-leukocyte adhesion, all accounting for the dramatic thrombi formation and organ dysfunction. Thus, inhibiting or preventing the formation of IL-1 avoids all these deleterious and potentially fatal effects; hence the recommendation for the use of *IL-1 receptor antagonist* (IL-1Ra) which can prevent hemodynamic changes, septic shock, organ inflammation and vascular thrombosis in patients with COVID-19 infection.^59^

## Post-Discharge Thromboprophylaxis

Data are emerging that patients hospitalized for COVID-19 infection may need post-discharge thromboprophylaxis. According to a retrospective observational cohort study of 163 COVID-19 patients who were discharged from the hospital without receiving anticoagulation, the cumulative incidence of thrombosis (including arterial and venous events) at day 30 following discharge was 2.5% (95% CI 0.8-7.6), of VTE 0.6% (95% CI 0.1-4.6) and of major hemorrhage 0.7% (95% CI 0.1-5.1) with clinically relevant non-major bleeds at 2.9% (95% CI 1.0-9.1).^60^

## Current Guidelines

Several societies have recently produced guidelines regarding the prophylactic and therapeutic management of VTE in patients with COVID-19 infection, which are outlined in Table 3.^5,61-67^ In addition, prior to the COVID-19 pandemic, the American Society of Hematology had issued guidelines (2018) for management of VTE for hospitalized and non-hospitalized medical patients.^68^ In that document, strong recommendations included use of VTE prophylaxis in acutely or critically ill inpatients at acceptable bleeding risk; use of mechanical prophylaxis in patients with high bleeding risk; advising against use of DOACs during hospitalization, and against extending pharmacological prophylaxis after hospital discharge. However, certain differences may apply for COVID-19 patients, as outlined in Table 3.

**Table 3.** Current Guidelines on Prophylactic and Therapeutic Intervention for Thromboembolism in Patients With COVID-19 Infection.

	Chinese Guidelines^61^	Italian Guidelines^62^	Swiss Guidelines^65^	International Guidelines^5^	American Guidelines^67^	ISTH Guidance^63^	Brazilian Guidelines^64^	Dutch Guidelines ^66^
Prophylaxis	VTE prophylaxis for all severe and critically ill COVID-19 pts in absence of contraindication For mild / moderate COVID-19 pts, determine VTE risk / VTE prevention in high- and moderate-risk pts in absence of contraindications For pts at high risk of bleeding or with active bleeding use IPC Use LMWH as first-line treatment / In pts with severe renal impairment (CrCl: <30mL/min), use UFH In case of thrombo-cytopenia with suspected HIT, use non-heparin anticoagulants (danaparoid, argatroban, or bivalirudin) over fondaparinux or rivaroxaban	Use of LMWH, UFH, or fondaparinux at doses indicated for VTE prophylaxis is strongly advised in all COVID-19 hospitalized pts Pts with contra-indications should be treated with limb compression Thrombo-prophylaxis should be given for the entire duration of the hospital stay and also maintained at home for 7-14d after discharge or in the pre-hospital phase, in case of pre-existing or persisting VTE risk factors	All in-hospital COVID-19 pts should receive thromboprophylaxis per risk stratification score, unless contraindicated In pts with CrCl >30 ml/min: LMWH / A higher dose in over-weight pts (>100 kg) In pts with CrCl <30 ml/min: UHF sc bid or tid or IV / A higher dose in overweight pts (>100 kg) Anti-Xa activity should be monitored when indicated (e.g., evidence of renal dysfunction) Antithrombin need not be monitored or consider on an individual basis in cases of DIC or sepsis-induced coagulopathy or heparin resistance Regularly monitor PT, D dimers, fibrinogen, platelet count, LDH, creatinine & ALT (daily or at least 2−3 x/w) In pts in ICU with a large increase in D dimers, severe inflammation, or signs of hepatic or renal dysfunction or imminent respiratory failure, intermediate or therapeutic dosing of LMWH or UHF should be considered, according to bleeding risk HIT should be considered in pts with fluctuations in platelet counts or signs of heparin resistance In pts with ECMO, maintain UFH at doses bringing anti-Xa activity into the therapeutic range There are no data on the use of DOACs	**-Pts with mild COVID-19 (outpatient)** Prophylaxis after risk assessment on individual basis for pts who have ↑ VTE risk, without high bleeding risk **-Pts with moderate / severe COVID-19 without DIC (hospitalized)** Thrombo-prophylaxis: enoxaparin 40 mg/d or similar LMWH (e.g., dalteparin 5,000 U/d) / SC heparin (5,000 U 2-3 x/d) for pts with renal dysfunction (i.e., CrCl<30 ml/min) If prophylaxis is contraindicated → IPC **-Pts with moderate or severe COVID-19 and DIC (hospitalized)** For pts without overt bleeding, prophylactic anticoagulation should be given For pts on chronic OAC, who develop DIC without bleeding, reasonable to consider anticoagulation and weigh with risk of bleeding when making clinical decisions regarding dose adjustments or discontinuation For pts with an indication for DAPT (e.g., PCI within 3 mos or recent MI) and DIC without overt bleeding, individualize decisions / In general, reasonable to continue DAPT if plt count is >50,000, reduce to single antiplatelet therapy if plt count is >25,000 and <50,000 and stop if plt count <25,000 For pts with COVID-19 being discharged, routine screening for VTE risk is reasonable for consideration of pharmacological prophylaxis for up to 45 days post-discharge	Thrombo-prophylaxis in acutely or critically ill hospitalized patients with COVID-19 using LMWH or fondaparinux over UFH; or LMWH, fondaparinux or UFH over DOAC Recommending against use of antiplatelet agents Recommending current standard dose anticoagulant thromboprophylaxis over intermediate (LMWH bid or increased weight-based dosing) or full treatment dosing Recommending inpatient thromboprophylaxis only over inpatient plus extended thromboprophylaxis after hospital discharge Suggesting against the addition of mechanical prophylaxis to pharmacological thromboprophylaxis, unless there is a contraindication to drugs	Monitor PT, D-dimer, platelet count, and fibrinogen Prophylactic dose LMWH in all patients who require hospital admission	Follow the WHO interim guidance statement: prophylactic LMWHs qd, or SC UFH bid * In case pharmacological prophylaxis is contraindicated: mechanical VTE prophylaxis (intermittent pneumatic compression) in immobilized patients Extended VTE prophylaxis should be considered after hospital discharge with enoxaparin or DOACs (up to 45 days) †	Prophylactic-LMWH should be initiated in all hospitalized pts with COVID-19, irrespective of risk scores Obtain a baseline (non-contrast) chest CT in all pts In pts with high clinical suspicion for PE, CTPA should be considered if D-dimer is ↑, i.e. ≥500 mg/L, age-adjusted threshold, or ≥1,000 mg/L when no YEARS criteria are present ** Obtain routine D-dimer testing on admission and serially during hospital stay with additional imaging as available In pts with a D-dimer <1,000 μg/L on admission and no significant ↑ during FU, prophylactic anticoagulation should be continued In pts with a D-dimer <1,000 μg/L on admission but a significant ↑ during hospital stay to levels above 2,000-4,000 μg/L, imaging for DVT or PE should be considered For pts with D-dimer between 1,000 and 2,000 μg/L, start prophylactic anticoagulation
Therapy	Parenteral **LMWH** as first line treatment in absence of contraindication In critically COVID-19 severe cases and signs of massive or high-risk PE, use rescue **thrombolysis** In refractory circulatory collapse or cardiac arrest, consider **ECMO** in combination with surgical **embolectomy** or catheter-directed treatment	Therapeutic doses of UFH or LMWH, only for established diagnoses of VTE or as a bridging strategy in pts on VKA In pts requiring therapeutic doses of LMWH or under DOAC, renal function should be monitored and anti-factor Xa or plasma DOAC levels should be tested Both VKA and DOAC have interference with antiviral treatment in COVID-19 pts / An individualized approach is recommended		Therapeutic anticoagulation for VTE / Parenteral anticoagulation (e.g., UFH) is preferred For agent selection consider comorbidities (renal or hepatic dysfunction, thrombocytopenia, and GI tract function) Concerns with UFH include the time to achieve therapeutic aPTT and increased health care worker exposure for frequent blood draws Thus, LMWHs may be preferred in pts unlikely to need procedures The benefit of OAC with DOACs includes no need for monitoring, facilitation of discharge planning, and outpatient management Potential risk: clinical deterioration and lack of timely availability of reversal agents For pts who are ready for discharge, DOACs or LMWH would be preferred Use of **catheter-directed therapies** should be limited to the most critical situations Selective use IVC filters In case of further deterioration, rescue systemic **fibrinolysis** should be considered, with catheter-directed options as an alternative. For patients with overt hemodynamic instability systemic **fibrinolysis** is indicated, with **catheter-based therapies** reserved for scenarios that are not suitable for systemic fibrinolysis. If infection control settings are equal, bedside initiation of **ECMO** is preferred	For acutely ill hospitalized COVID-19 pts with DVT or PE, use parenteral anticoagulation with LMWH or IV UFH In pts without any drug-to-drug interactions, use OAC with apixaban or rivaroxaban / Dabigatran and edoxaban can be used after initial parenteral anticoagulation / VKA can be used after overlap with initial parenteral anticoagulation For outpatients with proximal DVT or PE and no drug-to-drug interactions, use DOAC / Initial parenteral anticoagulation is needed before dabigatran and edoxaban / For pts who are not treated with a DOAC, use VKA over LMWH (for pt convenience and comfort) / Parenteral anticoagulation needs to be over-lapped with VKAs For COVID 19 pts with proximal DVT or PE, use anticoagulation for a minimum of 3 months Use thrombolysis for hemodynamically compromised pts without high risk of bleeding In pts with recurrent VTE despite anticoagulation with LMWH, ↑dose of LMWH by 25-30%		Full anticoagulation (mainly LMWH, ‡ fondaparinux, or UFH while at hospital and DOACs for long-term treatment) at standard doses ↓The use of IVC filters and catheter-directed thrombolysis Home treatment whenever possible Treat suspected PE as PE PE management should follow international guidelines Submassive but stable PE = anticoagulation Massive (unstable PE) = fibrinolysis No evidence to ↑ doses off-label Dose adjustments per renal function‡	If PE and/or DVT is confirmed, therapeutic anticoagulation is indicated

CrCl = creatinine clearance; DOACs = direct oral anticoagulants; ECMO = extracorporeal membrane oxygenation; HIT = heparin-induced thrombocytopenia; IPC = intermittent pneumatic compression; ISTH = International Society of Thrombosis and Hemostasis; IVC = inferior vena cava; LMWH = low-molecular weight heparin; pts = patients; UFH = unfractionated heparin; VKA = vitamin K antagonist; VTE = venous thromboembolism; WHO = World Health organization.

* Enoxaparin 40 mg SC once daily* / Fondaparinux 2.5 mg once daily /Unfractionated heparin 5.000 IU SC bid.

† ↓risk of VTE but ↑major bleeding.

‡ N.B.: Enoxaparin dose adjustment: CrCl < 30 mL/minute = enoxaparin 20 mg SC once daily (↓50% of the dose) / Enoxaparin dose adjustment for body mass index (BMI): 40-60 mg qd for BMI 30-40 kg/m^2^; 40 mg bid for BMI > 40 kg/m^2^; 60 mg bid for BMI > 50 kg/m^2^.

N.B.: DOAC doses for COVID-19 patients: 1) Rivaroxaban 15 mg bid x 3w→ 20 mg qd x 6 mos → 20 mg/10 mg qd extended; 2) Apixaban 10 mg bid x 1w → 5 mg bid x 6 mos → 2.5 mg bid extended; 3) Dabigatran: UFH/LMWH x 5d → dabigatran 150 mg bid x 6 mos / extended; 4) Edoxaban: UFH/LMWH x 5d → 60 mg to 30 mg qd x 6mos/extended.

** The YEARS clinical decision rule consists of 3 items (clinical signs of DVT, hemoptysis, and whether PE is the most likely diagnosis), and D-dimer levels.^51^

## Conclusion and Perspective

Ample evidence has recently been accumulated indicating that COVID-19 infection may induce a viral coagulopathy and a thrombotic cascade which finally leads to clinical manifestations of venous and arterial macro- and micro-thrombosis including DVT, PE or pulmonary arterial thrombosis, microvascular thrombosis, other arterial thromboses, acute myocardial infarction and ischemic stroke. Two phenotypic patterns of thrombotic manifestations of COVID-19 infection have been discerned, the classical *thromboembolic* disease, also observed in other types of sepsis, and the diffuse *micro-thrombotic* type, prevailing in the lungs but occasionally extending to other organs, as well. The molecular mechanisms implicated in this thrombotic state observed in patients with COVID-19 have not been fully elucidated; however, there appears to be a close link between inflammatory and hemostatic systems, involving infected endothelial cells, leukocytes and platelets, as well as complement activation and the hypoxic milieu produced by the virus which can further enhance these processes (Figure 1). There is both hypercoagulability and impaired thrombolysis that account for this viral coagulopathy. Medical societies have issued guidelines or consensus statements (Table 3) to guide clinicians in properly applying thromboprophylaxis and antithrombotic therapy in these patients both during the hospital stay and after discharge.

Importantly, all these data emanate mostly from observational studies and a lot need to be done to further investigate the extent of this crucial problem that has emerged as a major determinant of the clinical outcome of COVID-19 patients. We need to further scrutinize screening tools to early detect this prothrombotic state that will not be limited to coagulation markers but will also include markers of infection, inflammation and endothelial function (Table 2). We need to refine our diagnostic tools that determine the extent of lung injury and focus on microvascular thrombosis that could be helpful to guide individualized thromboprophylaxis and antithrombotic treatment for COVID-19 patients. Furthermore, we are in dire need for further evidence from RCTs of optimal anticoagulant and antithrombotic regimens that will effectively protect and manage this particular group of patients; current thromboprophylaxis schemes seem to be incomplete as they do not fully protect these patients from VTE.

Importantly, preliminary data indicate that, in addition to anticoagulants, other therapies need to be explored, such as anticomplement and NET inhibiting agents, IL-1 receptor antagonists, and other yet to be discovered agents, as they emerge from our better understanding of the pathogenetic mechanisms involved in this unusual and probably singular thrombotic process, which seems to differ from those processes that we, hitherto, have been familiar with.

## Abbreviations

ACE: angiotensin converting enzyme
ARDS: acute respiratory distress syndrome
COVID-19: Coronavirus-2019
CRP: C-reactive protein
CT: computed tomography
CTPA: computed tomography pulmonary angiography
DIC: disseminated intravascular coagulation
DVT: deep venous thrombosis
ESR: erythrocyte sedimentation rate
ICU: intensive care unit
IL: interleukin
MPV: mean platelet volume
NET: neutrophil extracellular traps
PE: pulmonary embolism
STEMI: ST-elevation myocardial infarction
TF: tissue factor
TxB2: thromboxane B2
VTE: venous thromboembolism
